# Exercise vs competitive athletics in youth: a neuroscience perspective

**DOI:** 10.3325/cmj.2015.56.581

**Published:** 2015-12

**Authors:** Lukasz M. Konopka

**Affiliations:** Spectrum Center for Integrative Neuroscience, McHenry, IL, USA lmkonopka@gmail.com

Today, basic and clinical science constantly declare the benefits of exercise, particularly, cardiovascular exercise. We hear that cardiovascular exercise benefits not only the cardiovascular system, but overall physiological and psychological health by increasing perfusion and advantageous biochemicals, such as brain-derived growth factors and neurotransmitters, and enhancing brain plasticity (1-3). Moreover, experts tell us that exercise increases learning and brain function and reduces anxiety and depression (4,5). Yet, just as many endeavors reach the point of diminishing returns, so exercise also has its limitations. We clearly see this result in the injured athletes, who have been expected to continuously perform at a competitive level. From grammar school through college, organized sports revolves around winning, and we support this notion telling our children that if they push themselves to excel, they will achieve that goal.

Although a commitment to competitive sports often means a family life that is pushed to the sidelines, we permit and tolerate this lifestyle because we hope our child will receive a university scholarship or a lucrative professional sports career. These forces act upon young athletes without reasonable checks and balances that consider their future physical and mental health. We often forget that grammar school and high school athletics encompass a very short period in a child's life. Very few children advance to sports careers at the college or professional levels. Admittedly, in the midst of competition, it is easy to forget that young athletes have most of their lives ahead of them and that competitive sports can negatively impact their future quality of life. Nevertheless, the emerging data show that when young athletes injure their joints, they often develop abnormal joint function and, potentially, the early onset of arthritis (6,7). Frequently, coaches and trainers suggest temporary interventions such as icing and/or anti-inflammatory agents after an injury, but sometimes these seemingly minor sports injuries can actually require more aggressive intervention or they become more serious pathology later in life.

One emerging concern is brain injury in athletes who have suffered concussions. Very recent research has discovered that concussions frequently occur in head-jarring sports such as boxing, soccer, rugby, football, or lacrosse, and that following a concussion, significant neurophysiological changes occur in the brain. These often present as transient or lasting episodes of confusion, headache, irritability, inattention, changes in sleep patterns, and loss of working memory (8-10). Nevertheless, when a concussion occurs in the heat of a game, it can be difficult for coaches to accurately determine whether a player should return to play or be removed from the competition. In addition, the desire to win tends to color the judgment of players, parents, and coaches. Yet, a player who is returned to the game is much more susceptible to additional, subsequent brain injury (11). Current data also argue that repeated concussions may have a cumulative effect on brain function (12,13). I have seen these consequences in my own practice. Yet, despite a large body of evidence, there are few well-designed methods for evaluating athletes after their injuries. There are also few attempts to modify games to limit or eliminate the possibility of injury.

Therefore, since education's primary goal is to enhance a child’s thinking ability and participation in life, we seem to hold completely irrational, self-defeating attitudes toward competitive sports: we struggle to develop better bodies and better minds for our athletes while simultaneously permitting their injury, thereby, crippling our attempts to create healthy, happy, educated citizens. It is time we opened a conversation about the risks of competitive athletics that undermine our ultimate goals and dreams for our children (14).
